# Latency period of PROM at term and the risk of neonatal infectious diseases

**DOI:** 10.1038/s41598-022-16593-6

**Published:** 2022-07-18

**Authors:** Lu Zhuang, Zhan-Kui Li, Yuan-Fang Zhu, Rong Ju, Shao-Dong Hua, Chun-Zhi Yu, Xing Li, Yan-Ping Zhang, Lei Li, Yan Yu, Wen Zeng, Jie Cui, Xin-Yu Chen, Jing-Ya Peng, Ting Li, Zhi-Chun Feng

**Affiliations:** 1 Faculty of Pediatrics, Chinese PLA General Hospital, Beijing, China; 2 Institute of Pediatrics, The Seventh Medical Center of Chinese PLA General Hospital, Beijing, China; 3 National Engineering Laboratory for Birth Defects Prevention and Control of Key Technology, Beijing, China; 4 Beijing Key Laboratory of Pediatric Organ Failure, Beijing, China; 5 Northwest Women’s and Children’s Hospital, Xi’an, Shanxi Province China; 6 Shenzhen Baoan Women’s and Children’s Hospital, Jinan University, Shenzhen, Guangdong Province China; 7 Chengdu Women’s and Children’s Central Hospital, School of Medicine, University of Electronic Science and Technology of China, Chengdu, China; 8 Department of Neonatology, BaYi Children’s Hospital, Seventh Medical Centre, PLA General Hospital, Beijing, 100007 China

**Keywords:** Risk factors, Infectious diseases, Epidemiology, Paediatric research

## Abstract

To find the risk of time thresholds of PROM for infectious diseases of term neonates. A multi-center prospective cohort study including pregnancies with PROM at term with a single fetus were conducted. Time thresholds of the duration from PROM to delivery were examined in 2-h increments to assess the rates of infectious neonatal diseases. 7019 pregnancies were included in the study. Neonatal pneumonia and sepsis were most frequent infectious diseases in neonates born from mother with PROM at term. Rates of early-onset pneumonia varied significantly when comparing length of time of PROM greater than 16 h vs. less than 16 h (for EOP in 3 days of life, adjusted OR 1.864, 95% CI 1.159 ~ 2.997, *p* = 0.010; for EOP in 7 days of life, adjusted OR 1.704, 95% CI 1.104 ~ 2.628, *p* = 0.016). Neonates born from mother of whom the length of time from PROM to delivery ≥ 16 h were at a higher risk of acquiring EOP.

## Introduction

Prelabor rupture of membranes (PROM), previously known as premature rupture of membranes^1^, occurs in approximately 8% ~ 18% pregnancies^2,3^. Pregnancies with PROM were at higher risk of intrauterine infection^2^, their neonates were at higher risk of infectious diseases^3^.

To identify those neonates who are at risk of infection was essential. The signs of neonatal infection can be very subtle and difficult to differentiate from other conditions, especially during the early stages, and clinical deterioration can occur very rapidly^4^. Delay in initiating antibiotic treatment when it is needed may significantly increase neonatal diseases and mortality^4^. To determine the risk accurately is also important because prophylactic use of antibiotics might result in antibiotic treatment of many such infants who are not infected, and may lead to complications of antibiotic therapy (e.g. childhood asthma, allergy and obesity, and infant gut microbiota aberrancies)^5–9^. The current status of antibiotic usage is highly variable and often dependent on hospital preferences and personal experience even in hospitals of developed countries^10^.

It is controversial whether prophylactic antibiotics should be used in term neonates of mothers with PROM. Prolonged rupture of membranes is reported as a risk factor for perinatally acquired bacterial infection in term neonate^4,11^. Some clinicians start the usage of prophylactic antibiotics if rupture of membranes for ≥ 24 h^12,13^. Later, one study by Linder et al. suggested that it may be unnecessary to administer prophylactic antibiotics to term^14^, and this inference is only based on the result of early-onset sepsis (EOS). However, in NICE clinical guideline on antibiotics for the prevention and treatment of early-onset neonatal infection, PROM was classified as “non-red flag” as clinical indicators and risk factors for recognizing the septic neonate, and antibiotic treatment in the neonate was suggested if one “red flag” or more than one “non-red flag” risk factor or clinical indicator is present^15^. Although it is well known that risk of neonatal infectious diseases increases with increasing length of time of ruptured membranes^16,17^, time length from PROM to delivery was not considered as a factor for decision of antibiotic treatment.

However, most of the studies focused on EOS in preterm neonates or maternal infectious diseases and few studies aimed other infectious diseases in term neonates^17–19^. In Seaward’s study^16^, neonatal infection was assessed and time intervals were set to 24 h. In Andreas’s study, EOS was assessed and time intervals were set to 6 h. However, these studies may not accurately reflect the a priori risk of developing infectious diseases related to duration of membrane rupture until delivery.

In order to find out a more accurate time point of the increased risk of infectious neonatal diseases due to prolonged PROM, we did a secondary analysis of the cohort (MCPPNC, Multi-center Cohort of Pregnancies with PROM and their Neonates in China) and we would assess the rates of infectious neonatal diseases by bivariate and multivariable analysis using dichotomized time thresholds of length of PROM before delivery in 2-h increments.

## Methods

This is a secondary analysis of the previous cohort (MCPPNC, Multi-center Cohort of Pregnancies with PROM and their Neonates in China), a prospective, multi-center cohort study aimed to describe the epidemiology of PROM and assess the influence of the implementation of the guideline^3^.

The definition of PROM is rupture of membranes before the on-set of labor^20^. PROM was confirmed by pooling and positive PROM test (PH test or insulin-like growth factor binding protein 1 detection test). Briefly, participants were recruited from patients admitted the three participating medical centers in China with a diagnosis of PROM between August 1, 2017, to March 31, 2018. All of the women with PROM were included in the study and participants at an estimated gestational age (GA) of < 24 weeks and ≥ 42 weeks were excluded. Pregnancies without PROM were eligible for the inclusion of unexposed group (non-PROM Group) if they satisfied the following conditions: the same gestational week, admission date ± 3 days and age ± 5 years compared with recruited PROM pregnancies. Maternal and neonatal data were collected until 7 days (death or hospital discharge if hospitalized for no more than 7 days). Clinical data including demographic, pregnancy history, obstetric and neonatal treatment regiments, laboratory test results and diagnosis were collected. This study was approved by the Ethical Committee of PLA Army General Hospital, China (2017-42) and assigned on the Protocol Registration and Results System of ClinicalTrials.gov (NCT03251898).

In the present study, we included pregnancies with PROM at term (estimated GA ≥ 37 weeks from MCPPNC) with a single fetus.

The rates of common neonatal infectious diseases including neonatal pneumonia, neonatal sepsis, omphalitis of newborn, neonatal urinary tract infection, congenital syphilis, neonatal conjunctivitis or dacryocystitis, necrotising enterocolitis of newborn, pyogenic abscess of the skin, congenital cytomegalovirus infection, bacterial meningitis, fungal infection of fetus or newborn, Rotavirus infection of the neonates were calculated in our study. The definition of each above diseases and fetal distress was according to the ICD 11th revision.

As early-onset pneumonia which develops within the first week of life and early-onset sepsis (develops in the first 3 days) were supposed to be result from perinatal factors. The primary outcomes of our study were set to be early-onset pneumonia (EOP) in 3 days of life, early-onset pneumonia in 7 days of life and neonatal early-onset sepsis (EOS, neonatal sepsis at < 3 days of age).

EOS was defined by the presence of clinical symptoms and a positive culture from blood or cerebrospinal fluid samples drawn within 7 days of birth^21,22^. Neonatal pneumonia was confirmed if meet the criteria in all three categories: (1) If there is underlying pulmonary or cardiac disease, two serial X-rays demonstrating at least one of the following: New or progressive infiltrate, consolidation, cavitation pneumoatocele. If there is no underlying pulmonary or cardiac disease, one definitive imaging test result is acceptable; (2) Worsening gas exchange. Any of the following: O2 desaturation, increased oxygen requirement, increased ventilator demand; (3) Clinical/laboratory evidence. Must have at least three of the following: Temperature instability; Leukopenia (≤ 4000 WBC/mm^3^) or leukocytosis (≥ 15,000 WBC/mm^3^) and left shift (≥ 10% band forms); New onset of purulent sputum or change in character of sputum, or increased respiratory secretions or increased suctioning requirements; Apnea, tachypnea, nasal flaring with retractions of the chest wall or nasal flaring with grunting; Wheezing, rales, or rhonchi; Cough; Bradycardia (< 100 beats/min) or tachycardia (> 170 beats/min). Early-onset pneumonia was defined as neonatal pneumonia develops within the first 7 days of life^23^.

The primary predictors were the length of time from PROM to birth, were examined before and after various dichotomized time thresholds by using 2-h time increments as a predictor of the 2 outcomes. For example, women delivering with a total duration of ruptured membranes of 8 h or less were compared with all those delivering with duration of ruptured membranes greater than 8 h. Similar comparisons were made for 8-, 10-, 12-, 14-, 16-, 18-, 20-, 22- and 24-h or more time thresholds to determine the threshold at which rates of each of the outcomes of interest achieved statistical significance (p < 0.05).

Education level of the pregnancies were defined as four levels: (1) Master's degree or above; (2) Bachelor’s degree; (3) Associate’s degree; (4) High school diploma or less.

If there was no indications for cesarean section, the following treatments were recommended by the guideline released in 2015 in China which was mainly referred to guidelines of the USA and Europe, and latest evidence-based medical evidences^24^. The GBS (Group B Streptococci) examination was by culture from vaginal or rectum swabs. For antibiotic usage according to the guideline, term pregnancies with clinical chorioamnionitis or a GBS (Group B Streptococci) positive result (no matter before or after admitted to the hospital) should receive antibiotics. If there was no GBS result or the GBS result is negative, those who had a fever of ≥ 38·0 °C or whose interval from PROM to delivery were ≥ 18 h should receive antibiotics. We defined the treatment follow the above procedure to be “using antibiotics according to the guideline” (Antibiotic). Induction of labor within 2 ~ 12 h after PROM is suggested for term pregnancies. During induction of labor with oxytocin, a sufficient period of adequate contractions (at least 12 ~ 18 h) should be allowed for the latent phase of labor to progress before diagnosing failed induction and moving to caesarean delivery. We defined the treatment follow the above procedure to be “induction of labor according to the guideline” (IL).

For umbilical care of the newborns, sterilize with 75% alcohol and keep dry for 2 times per day. For omphalitis care, the umbilical region should be cleaned with 3% hydrogen peroxide, disinfected with 95% alcohol and kept dry. For eye care, use 0.9% sodium chloride dipped in cotton swabs to wipe eye secretions for 2 times per day.

Power analysis were done using PASS 11. Data were analyzed by SAS (version 9.4). We provided descriptive statistics of obstetric and neonatal information. Continuous variables were summarized as mean (SD) or median (Q1 ~ Q3), and categorical variables were summarized as frequencies and proportions. Fisher's exact probability test and χ^2^ were used when appropriate for categorical variables. Multiple logistic models were conducted to assess the length of time from PROM to birth with EOP in 3 days of life, EOP in 7 days of life and EOS. We add the following potential confounding variables: the city where the hospital locates (sorted by latitude from low to high), the mother’s age, education level, induction of labor, prenatal antibiotic treatment, mode of delivery (the final way of delivery which means caesarean section or vaginal delivery), meconium-stained amniotic fluid (MSAF), the neonates’ sex, Apgar Score at 1 min (≤ 7 vs. ≥ 8).

### Statement of ethics

This study was approved by the Ethical Committee of PLA Army General Hospital, China (2017-42) and assigned on the Protocol Registration and Results System of ClinicalTrials.gov (NCT03251898). All participants provided written informed consent.

## Results

**Figure 1:**
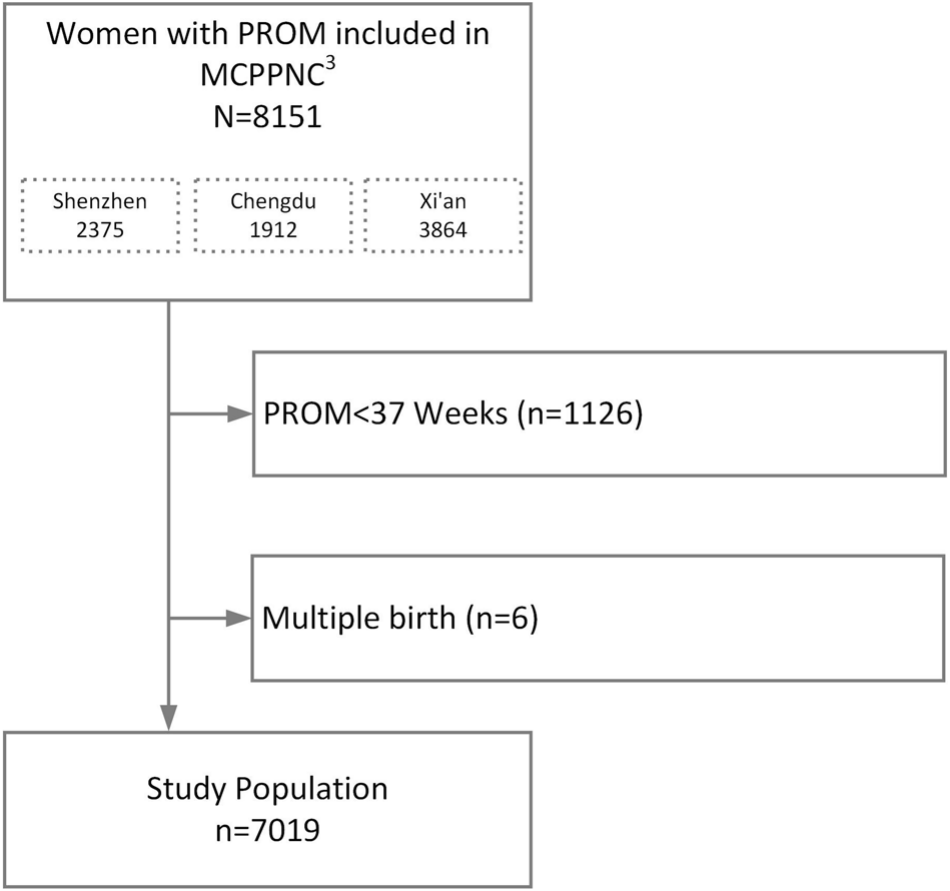
The flow chart summarizes how the sample size of the analysis was reached.

There were 7019 women who met inclusion criteria (Fig. 1). Demographic data and perinatal outcomes were showed in Table 1. The mean age of the pregnancies was 30.11 ± 4.00 and the mean gestational age was 38.81 ± 1.07 weeks. 29.25% (2053) of them were multiparous. 1634 pregnancies (23.28%) received induction of labor. The mean length of time from rupture of membrane to delivery were 20.52 ± 18.01 h. 272 (272/7019, 3.88%) fetuses were combined with fetal distress. Four fetuses (0.06%) died before birth and 7015 neonates were born. (Table 1).

**Table 1:** Maternal and neonatal characteristics among pregnancies with and without PROM.

Maternal	No. or mean ± SD	%
Sample size	7019	
Yellow race	7019	
Age (year), mean (SD)	30.11 ± 4.00	
Gestational age (weeks)	38.81 ± 1.07	
**Parity**		
Primiparous	4966	70.75%
Multiparous	2053	29.25%
**Onset of labor**		
Spontaneous	3711	52.87%
Induced	1634	23.28%
Cesarean	1674	23.85%
Time from PROM to delivery (hours), median (Q1, Q3)	20.52 (8.57, 26.52)	
Stillbirth	4	0.06%
**Neonatal**		
Total number	7015	
Gender		
Female	3375	48.11%
Male	3640	51.86%
Apgar score		
≤ 7 at 1 min	26	0.37%
≤ 7 at 5 min	3	0.04%
≤ 7 at 10 min	1	0.01%

*GA* gestational age, *Q1* quartile 1, *Q3* quartile 3.

Percentages were tested with a χ^2^ test. Medians were tested with a Wilcoxon rank sum test.

^a^*P* value is significant at α = 0.05 level of significance.

Totally 4169 (59.40%) pregnancies recieved antibiotics before birth. The reasons of mothers with PROM given prenatal antibiotics including: term pregnancies with clinical chorioamnionitis or a GBS (Group B Streptococci) positive result (no matter before or after admitted to the hospital) should receive antibiotics; if there was no GBS result or the GBS result is negative, those who had a fever of ≥ 38·0 °C or whose interval from PROM to delivery were ≥ 18 h should receive antibiotics; pregnancies who received cesarean section.

The rates of common neonatal infectious diseases were calculated. Generally, neonatal pneumonia was accounted for 1.71% (120/7019) and neonatal sepsis was accounted for 0.21% (15/7019). One of the neonates suffered from omphalitis and 2 of the neonates got urinary tract infection. Three of the neonates got conjunctivitis or dacryocystitis and 1 of the neonates was infected by Rotavirus. No other infections were found in our study.

The overall incidence of EOP in 3 days of life, EOP in 7 days of life and EOS was 1.45% (102/7019), 1.71% (120/7019) and 0.19% (13/7019), respectively. The coexistence of EOS and EOP in 3 days of life was 0.11% (8/7019). For EOS and EOP in 7 days of life, the coexistense was 0.13% (9/7019). The culture results of the EOS neonates were: *Staphylococcus hominis* (1), *Staphylococcus hominis* (1), *Listeria monocytogenes* (2), *Enterococcus faecalis* (1), *Klebsiella pneumonia* (1), *Escherichia coli* (2), *Staphylococcus epidermidis* (4), *Candida albicans* (1).

**Figure 2:**
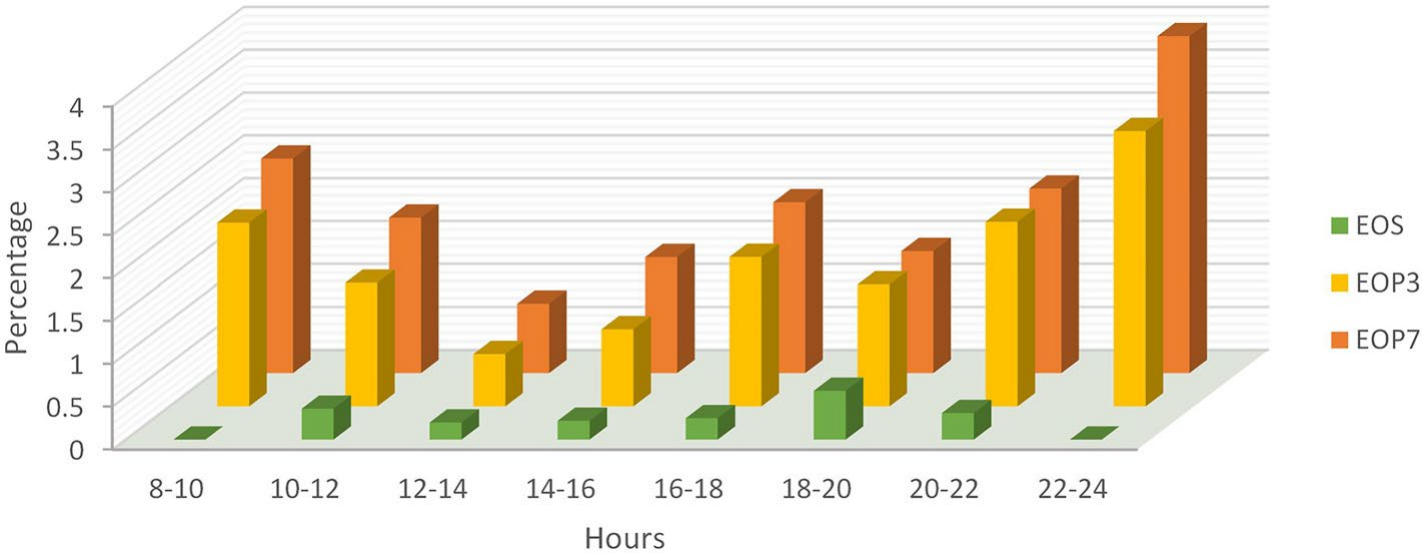
Length of time from rupture of membranes to delivery is categorized into 2-h groups and rates of EOP in 3 days of life, EOP in 7 days of life and EOS.

Figure 2 shows the neonatal infection rates by time of PROM. Rates of the outcomes were not noted to increase with increasing of time of ruptured membranes before delivery. There was a fluctuation of infectious neonatal relevant outcomes in a long (0–24 h) interval of PROM. The rates fluctuated over time (Fig. 2).

Various dichotomized time thresholds of total length of PROM as a predictor for EOP in 3 days of life, EOP in 7 days of life and EOS were examined in the multivariable model (Tables 2, 3, 4). After adjusted with the city where the hospital locates (sorted by latitude from low to high), the mother’s age, education level, induction of labor, prenatal antibiotic treatment, mode of delivery (the final way of delivery which means caesarean section or vaginal delivery), meconium-stained amniotic fluid (MSAF), the results showed that the rates of EOP in 3 days differed significantly at a time threshold of greater than 16 h vs. less than 16 h of PROM (adjusted OR 1.864, 95% CI 1.159 ~ 2.997, *p* = 0.0102), and this finding continued for subsequent 2-h increment thresholds examined until 20 h. For EOP in 7 days of life, the rates was also differed significantly since the time threshold of greater than 16 hous vs. less than 16 h of PROM (adjusted OR 1.704, 95% CI 1.104 ~ 2.628, *p* = 0.016). The multivariable analyses showed that there was no significant difference of early-onset sepsis when comparing length of PROM at any thresholds. At the conventional 2-tailed significance level of *P* = 0.05, and based on the sample size, rate at different time point, the power to show the OR quantifying the association between latency period from RPOM to birth and EOP and EOS are in Tables 2, 3, and 4.

**Table 2:** Multivariable analysis of length of time of PROM and neonatal outcomes: adjusted OR of other factors at time thresholds with significant differences of early-onset pneumonia in 3 days according to length of time of PROM.

Time threshold of PROM(h)^a^	Number of neonates	Rates of early onset pneumonia in 3 days	Early-onset pneumonia in 3 days OR [95% CI]	*P* value	Power
≥ 10	4882	1.54%	1.301 [0.770, 2.197]	0.3251	0.2051
≥ 12	4328	1.55%	1.283 [0.782, 2.106]	0.3245	0.2186
≥ 14	3833	1.67%	1.585 [0.974, 2.579]	0.0638	0.6343
≥ 16	3388	1.77%	1.864 [1.159, 2.997]	0.0102	0.8973
≥ 18	2986	1.78%	1.801 [1.137, 2.854]	0.0122	0.8667
≥ 20	2634	1.83%	1.789 [1.137, 2.815]	0.0118	0.8527
≥ 22	2309	1.78%	1.562 [0.995, 2.452]	0.0528	0.6211

Controlling for the city where the hospital locates, the mother’s age, education level, chorioamnionitis, induction of labor, prenatal antibiotic treatment, mode of delivery, amniotic fluid pollution, neonate’s sex, Apgar Score.

^a^Length of time of PROM from 0 h through specific time threshold.

**Table 3:** Multivariable analysis of length of time of PROM and neonatal outcomes: Adjusted OR of other factors at time thresholds with significant differences of early-onset pneumonia in 7 days according to length of time of PROM.

Time threshold of PROM(h)^a^	Number of neonates	Rates of early onset pneumonia in 7 days	Early-onset pneumonia in 7 days, OR [95% CI]	*P* value	Power
≥ 10	4882	1.78%	1.291 [0.803, 2.076]	0.2915	0.2309
≥ 12	4328	1.78%	1.256 [0.799, 1.974]	0.3240	0.2225
≥ 14	3833	1.91%	1.532 [0.981, 2.391]	0.0606	0.6547
≥ 16	3388	1.98%	1.704 [1.104, 2.628]	0.0160	0.8578
≥ 18	2986	1.98%	1.683 [1.103, 2.570]	0.0159	0.8468
≥ 20	2634	2.05%	1.747 [1.149, 2.657]	0.0090	0.8858
≥ 22	2309	2.04%	1.613 [1.061, 2.450]	0.0252	0.7543

Controlling for the city where the hospital locates, the mother’s age, education level, chorioamnionitis, induction of labor, prenatal antibiotic treatment, mode of delivery, amniotic fluid pollution, neonate’s sex, Apgar Score.

^a^Length of time of PROM from 0 h through specific time threshold.

**Table 4:** Multivariable analysis of length of time of PROM and neonatal outcomes: Adjusted OR of other factors at time thresholds with significant differences of early-onset sepsis according to length of time of PROM.

Time threshold of PROM(h)^a^	Number of neonates	Rates of early onset sepsis	Early-onset sepsis OR [95% CI]	*P* value	Power
≥ 10	4882	0.23%	3.279 [0.651, 16.518 ]	0.1499	0.3448
≥ 12	4328	0.21%	1.979 [0.501, 7.810]	0.3300	0.2116
≥ 14	3833	0.21%	1.991 [0.514, 7.708]	0.3189	0.2581
≥ 16	3388	0.21%	1.900 [0.510, 7.083]	0.3390	0.2558
≥ 18	2986	0.20%	1.736 [0.477, 6.318]	0.4029	0.2046
≥ 20	2634	0.15%	0.968 [0.255, 3.674]	0.9624	0.0495
≥ 22	2309	0.13%	0.774 [0.188, 3.178]	0.7220	0.0607

Controlling for the city where the hospital locates, the mother’s age, education level, chorioamnionitis, induction of labor, prenatal antibiotic treatment, mode of delivery, amniotic fluid pollution, neonate’s sex, Apgar Score.

^a^Length of time of PROM from 0 h through specific time threshold.

Tables 5 and 6 shows the adjusted OR of other factors at time thresholds with significant differences of the three outcomes according to length of time of PROM. For the outcome of EOP in 3 days and 7 days, the city where the hospitals locate showed great effect. Neonates whose Apgar score ≤ 7 showed to be at great risk of early onset Pneumonia.

**Table 5:** Adjusted OR of other factors at time thresholds with significant differences of EOP in 3 days according to length of time of PROM.

Time threshold of PROM(h)^a^	City	Mother’s age	Education			Induction of labor	Antibiotic	Caesarean section	MSAF	Child_sex	Apgar score ≤ 7
			BD	AD	HL						
16	0.523^b^ (0.392, 0.696)	0.967 (0.916, 1.021)	0.688 (0.315, 1.504)	0.505 (0.219, 1.167)	1.356 (0.594, 3.098)	0.154^b^ (0.070, 0.340)	1.798^b^ (1.084, 2.981)	1.427 (0.924, 2.204)	1.348 (0.811, 2.241)	0.905 (0.599, 1.369)	77.830^b^ (32.751,184.957 )
18	0.518^b^ (0.388, 0.690)	0.967 (0.915, 1.021)	0.697 (0.319, 1.523)	0.509 (0.220, 1.175)	1.374 (0.601, 3.140)	0.151^b^ (0.068, 0.335)	1.872^b^ (1.137, 3.082)	1.377 (0.894, 2.122)	1.351 (0.814, 2.245)	0.898 (0.594, 1.357)	78.508^b^ (33.157 185.892)
20	0.516^b^ (0.387, 0.689)	0.966 (0.915, 1.020)	0.695 (0.318, 1.520)	0.508 (0.220, 1.173)	1.354 (0.593, 3.094)	0.150^b^ (0.067, 0.333)	1.922^b^ (1.174, 3.146)	1.342 (0.872, 2.065)	1.341 (0.807, 2.228)	0.898 (0.594, 1.357)	78.383^b^ (33.062 185.826)
22	0.519^b^ (0.390, 0.692)	0.964 (0.913, 1.017)	0.684 (0.313, 1.493)	0.500 (0.217, 1.153)	1.334 (0.585, 3.041)	0.156^b^ (0.070, 0.346)	2.058^b^ (1.267, 3.343)	1.322 (0.859, 2.035)	1.345 (0.810, 2.235)	0.899 (0.595, 1.359)	74.734^b^ (31.706 176.154)

^a^Length of time of ruptured membranes from 0 h through specific time threshold.

^b^*P* < 0.05.

**Table 6:** Adjusted OR of other factors at time thresholds with significant differences of EOP in 7 days according to length of time of PROM.

Time threshold of PROM(h)^a^	City	Mother’s age	Education			Induction of labor	Antibiotic	Caesarean section	MSAF	Child_sex	Apgar score ≤ 8
			BD	AD	HL						
16	0.523^b^ (0.392, 0.696)	0.967 (0.916, 1.021)	0.688 (0.315, 1.504)	0.505 (0.219, 1.167)	1.356 (0.594, 3.098)	0.154^b^ (0.070 0.340)	1.798^b^ (1.084, 2.981)	1.427 (0.924, 2.204)	1.348 (0.811, 2.241)	0.905 (0.599, 1.369)	77.830^b^ (32.751 184.957)
18	0.518^b^ (0.388, 0.690)	0.967 (0.915, 1.021)	0.697 (0.319, 1.523)	0.509 (0.220, 1.175)	1.374 (0.601, 3.140)	0.151^b^ (0.068 0.335)	1.872^b^ (1.137, 3.082)	1.377 (0.894, 2.122)	1.351 (0.814, 2.245)	0.898 (0.594, 1.357)	78.508^b^ (33.157 185.892)
20	0.516^b^ (0.387, 0.689)	0.966 (0.915, 1.020)	0.695 (0.318, 1.520)	0.508 (0.220, 1.173)	1.354 (0.593, 3.094)	0.150^b^ (0.067, 0.333)	1.922^b^ (1.174, 3.146)	1.342 (0.872, 2.065)	1.341 (0.807, 2.228)	0.898 (0.594, 1.357)	78.383^b^ (33.062 185.826)
22	0.519^b^ (0.390, 0.692)	0.964 (0.913, 1.017)	0.684 (0.313, 1.493)	0.500 (0.217, 1.153)	1.334 (0.585, 3.041)	0.156^b^ (0.070, 0.346)	2.058^b^ (1.267, 3.343)	1.322 (0.859, 2.035)	1.345 (0.810, 2.235)	0.899 (0.595, 1.359)	74.734^b^ (31.706 176.154)

^a^Length of time of ruptured membranes from 0 h through specific time threshold.

^b^*P* < 0.05.

## Discussion

Our study was a real-world study on the relationship between infectious neonatal morbidity and mortality and latency period from PROM to delivery. We examined the relationship between the infectious diseases of neonates in NICU and the length of time from PROM to delivery by dichotomizing time intervals. We found the risk for EOP in 3 days of life and EOP in 7 days of life increased with increasing time of ruptured membranes since 16- hours.

There were few results on the relationship between infectious neonatal diseases and the length of time from PROM to delivery. The large International Multicenter Cohort Term PROM Study by Seaward et al.^16^ reported that ≥ 24 h was a risk factor of neonatal infection. A large cohort enrolled 113,568 singleton infants born at term in 2007 by Andreas et al.^17^ found that the risk of neonatal sepsis increased independently and nearly linearly with duration of membrane rupture up to 36 h, with an odds ratio of 1.29 for each 6-h increase in membrane rupture duration. A latest study by Shruti et al.^25^. reported that the rate of neonatal sepsis increases dramatically beyond 37 h of latency in term or near-term neonates (34 weeks ≤ GA ≤ 40 weeks) but the sample size was only 200. There is limited evidence of a more accurate time point of the increased risk of infectious neonatal diseases due to prolonged PROM. No evidence for EOP was reported.

In our study which enrolled pregnancies with PROM at term (estimated GA ≥ 37 weeks from MCPPNC) with a single fetus, the overall incidences of EOP and EOS in neonates werefound as 1.71% (120/7019) and 0.19% (13/7019). Within neonates admitted to NICU, early onset pneumonia in 3 days was 8/59(13.5%), early onset pneumonia in 7 days was 9/59(15.3%). It is reported acquired pneumonia was 21.3% (94/441) in neonates admitted NICU and 54.2% (totally 10.7%, 47/441) of them were early-onset pneumonia^26^. While in our study, within neonates admitted to NICU, early onset pneumonia in 3 days was 8/59(13.5%). The incidence of culture-proven early-onset neonatal sepsis in the United States is estimated to be 0.77 to 1 per 1,000 live births^27^. Among infants born at 37 weeks’ gestation or more in the United States, the rate of all-cause EOS is ~ 1.1 per 1000 LB (live birth) in black infants and ~ 0.4 per 1000 LB in nonblack infants^28^. In our previous study^3^, the rates of EOP and EOS in term neonates in PROM was significantly higher than those of non-PROM Group was significantly different. The result hints that for neonates born from mother with PROM at term, neonatal pneumonia and sepsis were main infectious diseases that need to be prevented and deal with.

For EOP in 3 days of life and EOP in 7 days of life, the city where the hospitals locate showed great effect. As in our study, different city means different hospitals, the difference may be related to the treatment strategies of different hospitals. Although there was recommendations by the Society of Obstetrics and Gynecology, Chinese Medical Association^24^, not all of the pregnancies were treated strictly according to the guidelines^3^. The difference of treatments might exist in different hospitals from different areas of our study. Other factors, such as mother’s age and education level, meconium-stained amniotic fluid, and neonate’s sex were not significant. The protective effect of induced labor is very significant. Curiously, use of antibiotics before delivery was a risk factor of EOP in 3 days of life and EOP in 7 days of life and this may because that mother who use antibiotics themselves were at higher risk of infection.

1-min Apgar Score (≤ 7) appeared to be a risk factor of early-onset pneumonia consistent with previous results. Elisha Ernest et al. reported that newborns, of any gestational age, with low 5 min Apgar scores appear to be at an increased risk for pediatric respiratory morbidity^29^. Sandra Costa et al. found that low Apgar score at one and five minutes was associated with neonates’ transient tachypnea, pneumonia^30^. Although apgar score cannot be used to decide whether resuscitation is needed, how to resuscitate and how to resuscitate, it has certain guiding significance for whether resuscitation should be continued. According to *Guidelines for Neonatal Resuscitation in China (2016)*^31^, the way of neonatal resuscitation including keeping warm, maintaining body position, clearing the airway, positive pressure ventilation and even endotracheal intubation et al. Neonatal tracheal lumen stenosis, immaturity of lung elastic fibers and other reasons make it easy for bacteria to invade the alveoli, trachea, bronchi and other parts. In addition, the introduction of exogenous microorganisms and mucosal damage during the process of clearing the airway and endotracheal intubation during resuscitation will also increase the possibility of infectious pneumonia.

Previous studies have evaluated 6^17^, 12^25^, or 24^16^ h cutoffs of latency period from PROM to delivery for term neonates. Most of the studies focused on neonatal sepsis. In addition, those methodology may not accurately reflect the priori risk of developing infectious diseases related to duration of PROM until delivery. There were limited evidences of a more accurate time point of the increased risk of infectious neonatal diseases due to prolonged PROM. No evidence for EOP was reported.

The limitation of our study was that we did not exclude neonates with critical congenital heart disease for that underlying cardiac disease might have effects on pneumonia. Critical congenital heart disease should be taken into account in the future study.

Our data was from real-world observations. Our study was strengthened by the sample size (7019) and being conducted at 3 centers from 3 different provinces of China and “city” as a confounder was also included in multivariable analysis. All pregnancies with PROM at term from MCPPNC which included all PROM pregnancies (24 weeks ≤ estimated gestational age (GA) ≤ 42 weeks) with a single fetus to avoid selective bias. Despite the considerable sample size of our study, one of the limitations was that the number of EOS cases were not sufficient to assess the influence of length of time from PROM to birth and chorioamnionitis.

## Conclusion

According to the data, the length of time from PROM to delivery ≥ 16 h is associated with an increased risk of EOP in 3 days of life and EOP in 7 days of life. The results could be a reference for antibiotic use of neonates born from mother with PROM.

## Data Availability

After publication, the data will be made available to others on reasonable requests to the corresponding author. A proposal with detailed description of study objectives and statistical analysis plan will be needed for evaluation of the reasonability of requests. Additional materials might also be required during the process of evaluation. Deidentified participant data will be provided after approval from the corresponding author and Seventh Medical Centre, PLA general hospital.

## Abbreviations

PROM: Prelabor rupture of membranes
EOS: Early-onset sepsis
MCPPNC: Multi-center cohort of pregnancies with PROM and their neonates in China
GA: Gestational age
C-section: Caesarean section
GBS: Group B Streptococci
IL: Induction of labor according to the guideline
MSAF: Meconium-stained amniotic fluid
BD: Bachelor’s degree
AD: Associate’s degree
HL: High school or less
